# Deletion of the Mitochondrial Flavoprotein Apoptosis Inducing Factor (AIF) Induces β-Cell Apoptosis and Impairs β-Cell Mass

**DOI:** 10.1371/journal.pone.0004394

**Published:** 2009-02-06

**Authors:** Fabienne T. Schulthess, Sophie Katz, Amin Ardestani, Hiroshi Kawahira, Senta Georgia, Domenico Bosco, Anil Bhushan, Kathrin Maedler

**Affiliations:** 1 Centre for Biomolecular Interactions, University of Bremen, Bremen, Germany; 2 Larry L. Hillblom Islet Research Center, Department of Medicine, University of California Los Angeles, Los Angeles, California, United States of America; 3 Cell Isolation and Transplantation Center, Department of Surgery, University of Geneva School of Medicine, Genèva, Switzerland; Mayo Clinic College of Medicine, United States of America

## Abstract

**Background:**

Apoptosis is a hallmark of β-cell death in both type 1 and type 2 diabetes mellitus. Understanding how apoptosis contributes to β-cell turnover may lead to strategies to prevent progression of diabetes. A key mediator of apoptosis, mitochondrial function, and cell survival is apoptosis inducing factor (AIF). In the present study, we investigated the role of AIF on β-cell mass and survival using the Harlequin (*Hq*) mutant mice, which are hypomorphic for AIF.

**Methodology/Principal Findings:**

Immunohistochemical evaluation of pancreata from *Hq* mutant mice displayed much smaller islets compared to wild-type mice (WT). Analysis of β-cell mass in these mice revealed a greater than 4-fold reduction in β-cell mass together with an 8-fold increase in β-cell apoptosis. Analysis of cell cycle dynamics, using BrdU pulse as a marker for cells in S-phase, did not detect significant differences in the frequency of β-cells in S-phase. In contrast, double staining for phosphorylated Histone H3 and insulin showed a 3-fold increase in β-cells in the G2 phase in *Hq* mutant mice, but no differences in M-phase compared to WT mice. This suggests that the β-cells from *Hq* mutant mice are arrested in the G2 phase and are unlikely to complete the cell cycle. β-cells from Hq mutant mice display increased sensitivity to hydrogen peroxide-induced apoptosis, which was confirmed in human islets in which AIF was depleted by siRNA. AIF deficiency had no effect on glucose stimulated insulin secretion, but the impaired effect of hydrogen peroxide on β-cell function was potentiated.

**Conclusions/Significance:**

Our results indicate that AIF is essential for maintaining β-cell mass and for oxidative stress response. A decrease in the oxidative phosphorylation capacity may counteract the development of diabetes, despite its deleterious effects on β-cell survival.

## Introduction

Apoptosis of the insulin producing β-cells and the decline in β-cell mass is a major mechanism of the progression of diabetes [1]. In addition, the rapid loss of β-cell function and the relative discrepancy between insulin demand and insulin secretion worsen the ability of the organism to maintain normoglycemia.

Important metabolites that regulate insulin secretion are generated in the mitochondria [2], [3]. The β-cell mitochondria are key regulators of glucose stimulated insulin secretion, and several mitochondrial pathways are disabled in type 2 diabetes (T2DM), e.g. the glucose induced hyperpolarization of the mitochondrial membrane and the raise in the ATP/ADP ratio at high glucose. Diabetic islets also have increased UCP-2 expression, which uncouples mitochondrial oxidative phosphorylation, such that energy is wasted through heat, and cellular ATP synthesis is decreased, probably through an increased formation of reactive oxygen species (ROS) [4].

Besides regulating factors of insulin secretion, mitochondria play a prominent role in apoptosis. In β-cells, elevated free fatty acids and production of ROS have been shown to induce apoptosis through the release of cytochrome c from the inner mitochondrial membrane to the cytosol, which triggers caspase activation [5], [6]. Mitochondria are the principal source of ROS in the β-cell [7], which accumulates when the respiratory chain function is defective. The low expression of an antioxidant enzyme defense system makes the β-cells particularly susceptible to an oxidative insult [8].

The apoptosis-inducing factor (AIF) is a mitochondrial intermembrane protein, which translocates to the cytoplasm and nucleus during apoptosis [9], where it binds to DNA triggering DNA fragmentation and nuclear condensation [9], [10]. In contrast to cytochrome c, AIF induces apoptosis in a caspase-independent fashion [11]. AIF is essential for mammalian development, knocking out AIF in the mouse is lethal before birth [12]. Therefore, the role of AIF as a death executor or as an oxidative stress scavenger is controversial. Evidence that AIF serves as free radical scavenger to prevent apoptosis came from studies on the Harlequin (*Hq*) mutant mouse. These mice have an 80% reduction in AIF protein due to a proviral insertion in the first intron of the AIF gene. They exhibit a reduced oxidative phosphorylation (OxPHOS) in the retina and in the brain, which entails increased oxidative stress and neuronal cell death through unscheduled cell cycle reentry [13].

Defects in OxPHOS and mitochondrial energy metabolism have been implicated in human diseases, including insulin resistance [14], [15] and diabetes mellitus [16], neurodegenerative disorders such as Alzheimer's disease, Parkinson's disease, as well as aging [17], [18]. Given the different roles of AIF in maintaining a functional respiratory chain, protecting cells from oxidative stress, but also contributing to stress- induced cell-death and that important mitochondrial pathways control β-cell pathophysiology, we asked the question what role does AIF play in the regulation of β-cell survival, mass and insulin secretion?

## Materials and Methods

### Animal breeding and genotyping

Harlequin (Hq) arose on a CF1 outbred stock and was transferred to a BGCBACa-A^w-J^/A (B6CBA) background. The Hq mice were obtained from Jackson Laboratory (Bar Harbor, ME). For controls, WT littermates which do not have the Hq mutation (+/Y) were used. Animals were housed at 22°C with a 12-h light-dark cycle (lights on at 07:00) and allowed free access to water and chow according to the protocol approved by the UCLA Chancellor's Animal Research Committee in agreement to NIH animal care guidelines.

### Islet isolation and culture

Human islets from six different donors were obtained from the Islet cell resource center (ICR-ABCC) or the European Consortium for Islet Transplantation (ECIT). Mouse islets were isolated by bile duct perfusion and collagenase digestion as described before [5]. The islets were cultured in suspension culture dishes. Human islets were cultured in CMRL 1066 medium containing 5.5 mM glucose and mouse islets in RPMI 1640 medium containing 11.1 mM glucose, both supplemented with 100 U/ml penicillin, 100 µg/ml streptomycin and 10% FCS (Invitrogen Ltd., Carlsbad, CA), hereafter referred to as culture medium. Islets were pre-cultured for 48 h after the isolation before the experiment, in some experiments, islets were transfected with siRNA and/or treated with 50 µM H_2_O_2_ (Sigma) for 2 h.

### β-cell mass analysis and immunohistochemistry

At the age of 2 and 9 weeks of age, mice were sacrificed and pancreata dissected, weighed and fixed in formalin followed by paraffin embedding, orienting pancreata such that sections were cut along the head-tail axis. Sections for β-cell mass were at least 50 microns apart. Five representative sections from each pancreas (spanning the width of the pancreas) were used in the analysis of β-cell mass. Pancreatic and islet sections were deparaffinized in toluene, rehydrated in grades of alcohol and washed in H_2_O. Slides were exposed to antigen-retrieval using antigen unmasking buffer according to the manufacturer's instructions (Vector Laboratories, Inc. Burlingame, CA). After antigen unmasking, the sections were cooled to room temperature, permabilized in 0.4% Triton X-100/TBS for 30 min, and blocked with 0.2% Tween 20/3% IgG-free BSA/2% Triton X-100/TBS. Primary antibodies were diluted at the following dilutions: guinea pig anti-insulin 1∶50 (Dako, Carpinteria, CA), mouse anti-glucagon 1∶1000 (Sigma-Aldrich, St. Louis, MO), rabbit anti-phosphohistone H3 1∶200 (Upstate, Charlottesville, VA) followed by detection with donkey-and goat-derived secondary antibodies conjugated to FITC and Cy3 (Jackson ImmunoResearch Laboratories, West Grove, PA). A montage of the whole pancreatic section was created using OpenlabTM and ImageJ software (Improvisison, MA) on a Leica DM6000. The relative area of β-cells (green fluorescence) was determined by quantification of the cross-sectional β-cell area divided by the cross-sectional area of total tissue. β-cell mass per pancreas was estimated as the product of the relative cross-sectional area of β-cells per total tissue and the weight of the pancreas.

To obtain sections from isolated islets, islets were washed with PBS, fixed in Bouin's solution for 15 min. and resuspended in 2% melted agarose in PBS, followed by short centrifugation and paraffin embedding. 4 µM islet sections were cut and re-hydrated. For detection of β-cell apoptosis, sections were incubated with 20 µg/ml proteinase K (Roche Diagnostics, Indianapolis, IN) for 12 min at 37°C and blocked with 0.2% Tween 20/3% IgG-free BSA/2% Triton X-100/TBS. Apoptosis was analyzed by the terminal deoxynucleotidyl transferase-mediated dUTP nick-end labeling (TUNEL) technique according to the manufacturer's instructions (In Situ Cell Death Detection Kit, TMR red; Roche). Then, sections were double-stained for insulin as described above.

### Intra-peritoneal glucose tolerance test (IPGTT)

Mice were fasted for 16 h, baseline blood glucose levels were measured in tail-vein blood from mice using a Glucometer (Freestyle, TheraSense Inc, Alameda, CA). Glucose (2 mg/g body weight) was injected intraperitoneally and blood glucose was measured 15, 30, 60, and 120 min after injection.

### β-cell proliferation

5-bromo-2-deoxyuridine (BrdU) incorporation: BrdU was injected intraperitoneally (0.025 mg/g body weight) 2 h before harvesting the pancreata. Pancreata were isolated and processed for histology as described above. Mouse anti-BrdU antibody/nuclease solution (Amersham/Pharmacia) was applied for 1 h at RT and double staining for insulin was performed as described above.

### RNA extraction and quantitative RT-PCR analysis

Isolated islets were washed in cold PBS and homogenized in Tri Reagent (Molecular Research Center Inc.), and the total RNA was prepared according to the manufacturer's methods. For quantitative analysis, we used the LightCycler Quantitative PCR System (Roche) with a commercial kit (LightCycler FastStart DNA Master plus SYBR Green I, Roche). Primers used were: 5′-AGTGGAAGACTGGCTGGAGA-3′ and 5′-TCACTCTCCGAACGGATACC-3′ (mouse AIF); 5′-GTTGGCCAGGCTGGTGTCCAG-3′ and 5′-CTGTGATGAGCTGCTCAGGGTGG-3′ (tubulin).

### RNA interference

A knock down of AIF mRNA and protein levels was carried out using the small interfering RNA (siRNA) technique. The siRNA duplex was designed against the following target sequence: GCAACCTAGTGTACTTCTT synthesized by Dharmacon. The negative scramble siRNA #1 (Ambion, Austin, TX) was used as control. Transfection of cultured human islets with siAIF and control siRNA was performed by Lipofectamine2000 (Invitrogen) according to the manufacturer's instructions. Cell lysates were collected 72 h after transfection.

### Western blot analysis

Islets were washed in PBS and lysed for 40 min on ice in 40 µl lysis buffer containing 20 mM Tris acetate, 0.27 M sucrose, 1 mM EDTA, 1 mM EGTA, 50 mM NaF, 1% Triton X-100, 5 mM sodium pyrophosphate and 10 mM β-glycerophosphate. Prior to use, the lysis buffer was supplemented with Protease- and Phosphatase-inhibitors (Pierce, Rockford, IL). Equal amounts of protein of each treatment group were run on NuPAGE 4–12% Bis-Tris gels. After transfer onto PVDF filters, nonspecific binding sites were blocked for 1 h using 5% nonfat dry milk and 0.05% Tween 20 in Tris-buffered saline and incubated with rabbit anti-AIF 1∶1000 (Chemicon International, Temecula, CA) followed by horseradish-peroxidase-linked anti-rabbit IgG peroxidase. The emitted light was captured on X-ray film after adding Immobilon Western chemiluminescent HRP substrate (Millipore, Billerica, MA). To verify that equal amounts of protein were loaded, membranes were stripped and re-probed with rabbit anti-β-actin (Cell signaling, Beverly, MA).

### Glucose stimulated insulin secretion (GSIS)

After treatment, islets were washed and pre-incubated (30 min) in Kreb's Ringer bicarbonate buffer (KRB) containing 2.8 mM glucose and 0.5% BSA. KRB was then replaced by KRB 2.8 mM glucose for 1 h (basal), followed by an additional 1 h in KRB 16.7 mM glucose (stimulated). Islets were extracted with 0.18 N HCl in 70% ethanol for determination of insulin content. Islet insulin was determined using mouse insulin ELISA (ALPCO, Salem, NH).

### Statistical analysis

All data were expressed as means+/−SE. The statistical significance of differences was measured by Student's *t* test.

## Results

### AIF depletion decreases β-cell mass

**Figure 1:**
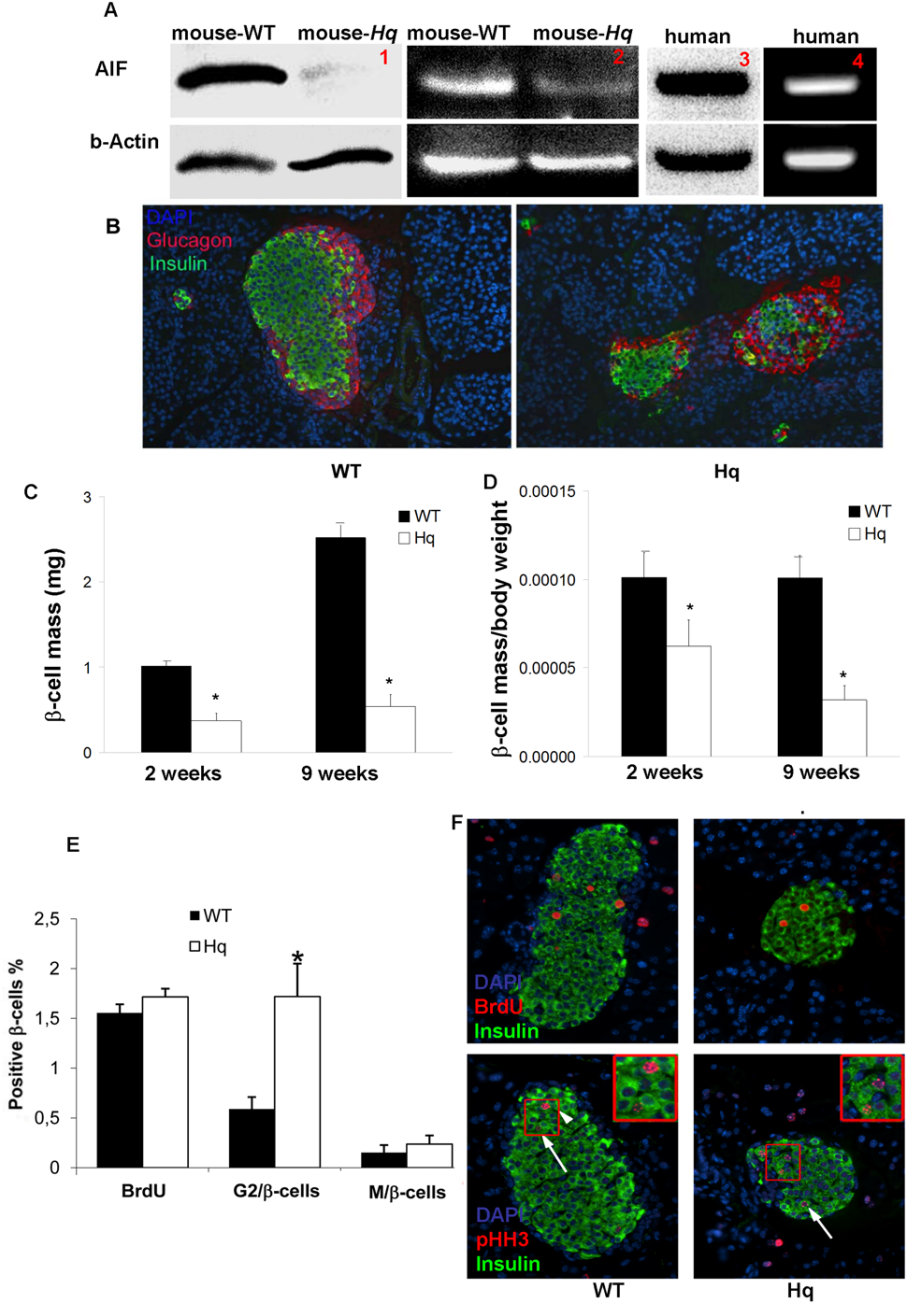
Decreased β-cell mass in Hq mutant mice. (A). Representative Western blots (panel 1,3) and PCR analyzes (panel 2,4) of AIF expression in isolated mouse (panel 1,2) and human (panel 3,4) islets. Actin was used as loading control/ house keeping gene. Western blots/ PCRs are representatives of three independent experiments from three mice or from 3 organ donors, respectively. (B) Histological analysis by insulin staining in green and glucagon staining in red show a normal islet cellular composition and smaller islets in 2-week-old *Hq* mutant mice. (C,D) Analysis of β-cell mass (C) or β-cell mass divided by body weight (D) of WT and *Hq* mutant mice at 2 and 9 weeks of age. Values are representative of 5 slides spanning the whole pancreas of each mouse and 4 mice for each group at each age (magnification x125). (E) Cell cycle characteristics of β-cells from WT mice and *Hq* mutant mice as measured by BrdU and pHH3 staining. BrdU^+^insulin^+^ cells are counted as β-cells at S phase (see example in F, upper panel). pHH3^+^ (with punctuated pattern) insulin+ cells are counted as β-cells at G2 phase (see example in F, Hq mice lower right panel). pHH3^+^ (with strong nuclear expression) insulin+ cells were counted as β-cells at M phase (see example in F, WT mice lower left panel). Data are shown as mean±SE. *P<0.05 in *Hq* mutant mice *vs*. WT mice. For section 1 of Figure 1A, the following copyright limitations apply: Copyright © 2006, American Diabetes Association. Published by American Diabetes Association. All rights reserved.

To understand if AIF plays a role in the regulation of survival in β-cells, we analyzed both mRNA and protein expression of AIF in isolated mouse and human pancreatic islets. RT-PCR analysis showed the mRNA transcript of AIF in both human and mouse islets. Western blot analysis confirmed the expression of AIF protein in isolated human and mouse islets (Fig. 1A). To elucidate if AIF played a role in the establishment of the endocrine pancreas, we compared the islet cellular composition of WT mice and *Hq* mutant mice, a hypomorphic AIF mouse model in which the expression of AIF is reduced to 10–20% of wild type littermates. This depletion of AIF was confirmed in isolated islets by RT-PCR and Western Blot analysis (Fig. 1A). Immunohistochemical evaluation of pancreata revealed normal islet morphology in the *Hq* mutant mice (Fig. 1B). Islet β-cell mass was 2.7-fold reduced in 2-week (p<0.001) and 4.6-fold in 9-week-old (p<0.01) *Hq* mutant mice, respectively compared to their WT littermates (Fig. 1C).

The average weight of a *Hq* pancreas was 53% of WT littermate pancreas at the age of 2 weeks and 60% at the age of 9 weeks (p<0.01). Normalization of the β-cell mass to whole body mass still resulted in a 39% and 68% decrease in the *Hq* mice at the age of 2 and 9 weeks, respectively. (Fig. 1D).

Possible reasons for the decreased β-cell mass could be increased β-cell apoptosis and/or reduced proliferation. Previous studies show that neurons from *Hq* mutant mice display an increased propensity to re-enter the cell cycle, which results in aberrant apoptotic destruction of the neurons [13]. To determine if cell cycle dynamics were misregulated in the *Hq* β-cells, we analyzed the incorporation/expression of cell cycle markers of β-cells in *Hq* mutant mice. A BrdU pulse was administered to 4 week old *Hq* and WT littermates 2 h before sacrifice to mark cells that were currently in S-phase. BrdU^+^insulin^+^ double-positive cells were counted as β-cells in S phase. Immunohistochemistry for phosphorylated Histone H3 (pHH3) staining allows one to discriminate cells in G2 or M phases of cell cycle [19], [20]. pHH3+insulin+ (with punctuated pattern) cells were counted as β-cells at G2 phase. pHH3+insulin+ (with a homogeneously condensed pattern of strong nuclear expression) cells were counted as β-cells at M phase (Fig. 1F). Analysis of BrdU incorporation did not reveal any significant difference in proliferating cells between WT and *Hq* mutant mice (Fig. 1E). However, pHH3 staining showed a 3-fold increase in β-cells in the G2 phase in *Hq* mutant mice but no differences in M-phase (Fig. 1E, *p*<0.05), an example of such punctuated staining pattern in the Hq mice is shown in Fig. 1F. This suggests that β-cells from *Hq* mutant mice either stop or prolong the G2 phase and some do not further re-enter the cell cycle. Previous studies show that such cells are highly prone to apoptosis [21], [22]. To test this possibility, we analyzed β-cell apoptosis in pancreatic sections from WT and *Hq* mutant mice at different ages. β-cell apoptosis in *Hq* mutant mice was increased at all ages compared to WT (Neonates P0: 0.4% in *Hq* mutant mice versus 0.2% in WT. No apoptosis was observed in the islets from WT mice at all further ages analyzed but the number of apoptotic β-cell in the *Hq* mutant mice were at P7: 0.3%, P14: 0.1%, 4-weeks-old: 0.2% and 9-weeks-old: 0.2%.)

### AIF depletion in mice leads to increased β-cell apoptosis

**Figure 2:**
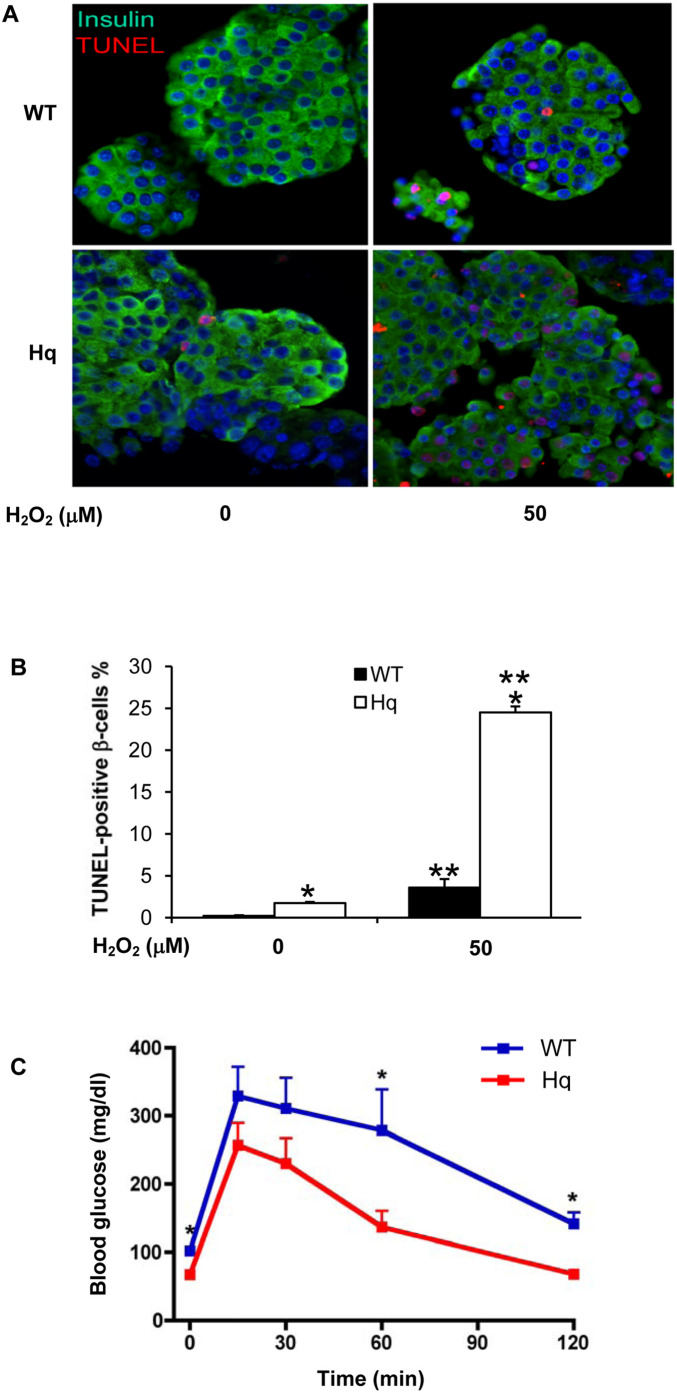
AIF depletion in mice leads to increased β-cell apoptosis. (A, B) Triple staining for TUNEL in red, insulin in green and 4′,6-diamidino-2-phenylindole (DAPI) in blue was performed on fixed, paraffin-embedded sections from isolated islets from 9-week old mice treated for 2 h with or without 50 µM H_2_O_2_. (B) Results are expressed as percentage of TUNEL-positive β-cells±SE. The mean number of β-cells counted was 700 for each treatment condition. (C) Glucose tolerance test with 2 mg/g BW glucose: Fasting and glucose stimulated plasma glucose levels are significantly lower in 12–16 week old *Hq* mutant mice (n = 9) compared to age-matched WT mice. Data are shown as mean + SE. *p<0.05 in *Hq* mutant mice vs. wt mice. **p<0.05 in H_2_O_2_ treated vs. untreated control.

Based on our *in vivo* results, we studied β-cell apoptosis from isolated islets in order to further investigate the nature of the β-cell loss in *Hq* mutant mice. Neurons [13] as well as cardiomycytes [23] from *Hq* mutant mice are highly susceptible to cell death induced by oxidative stress. Isolated mouse islets from mutant and WT animals were treated with 50 µM H_2_O_2_ for 2 h and β-cell apoptosis was analyzed by double staining for TUNEL and insulin. In accordance with our *in vivo* observations, β-cell apoptosis in the *Hq* mutant mice islets was 8-fold increased in the untreated islets (*p<0.05). When islets were exposed to H_2_O_2_, there was a 16.5-fold increase in β-cell apoptosis in the WT mice (**p<0,05), and a 14-fold increase in the *Hq* mutant mice islets (*p*<0.05). When compared to the WT mice, there was a 6.8-fold increase in the *Hq* mutant mice islets (*p*<0.05), when islets were exposed to H_2_O_2_ (Fig. 2A&B). Because apoptosis results in decreased β-cell mass, we hypothesized that *Hq* animals would be hyperglycemic. Contrary to our predictions, *Hq* mutant mice showed significantly lower fasting blood glucose levels as well as improved glucose tolerance during an ipGTT (Fig. 2C). Our data confirm previously published data that AIF depletion in the liver and/or skeletal muscle leads to increased insulin sensitivity [15]. Because *Hq* mice are global hypomorphs, we can only assume that minimal insulin secretion from the reduced β-cell mass is sufficient to maintain glucose homeostasis in insulin hyper-sensitive mice. This indicates that hepatic and muscular defects in the oxidative phosphorylation may counteract the development of diabetes, despite its deleterious effects on β-cell survival.

### AIF depletion leads to increased β-cell apoptosis in human islets without affecting insulin secretion

**Figure 3:**
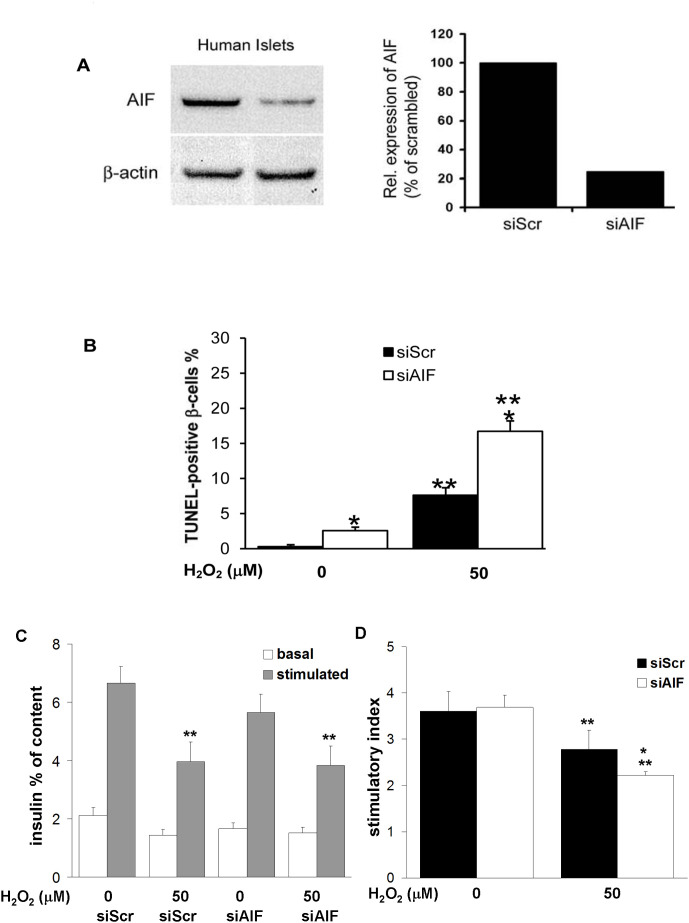
AIF depletion leads to increased β-cell apoptosis in human islets without affecting insulin secretion. (A) Isolated human pancreatic islets were exposed to siRNA to AIF (siAIF) or scrambled control siRNA (siScr) for 3 days. The knockdown efficiency was determined by Western blot analysis. Actin was used as a loading control on the same membrane after stripping. This Western blot is representative of three independent experiments from three different organ donors. The density of expression levels were quantified after scanning and normalised to actin levels. (B–D) 3 days after transfection, islets were exposed to 50 µM H_2_O_2_ for 2 h. (B) Islet sections were prepared for analysis of β-cell apoptosis by the TUNEL assay. Islets were double-stained for insulin in green and counterstained for DAPI in blue. Results are means±SE of the percentage of TUNEL-positive β-cells. The mean number of β-cells counted was 3400 for each treatment condition. (C,D) GSIS: after the H_2_O_2_ treatment, islets were washed and basal and stimulated insulin secretion analyzed during successive 1-h incubations at 2.8 mM (basal) and 16.7 mM (stimulated) glucose. Data are normalized to insulin content. (D) Stimulatory index denotes the ratio between stimulated and basal values of insulin secretion. (B–D) All assays were performed in triplicate or quadruplicate in three independent experiments from 3 different organ donors, respectively. *p<0.05 to siScr control, **p<0.05 in H_2_O_2_ treated vs. untreated control.

To investigate the significance of AIF and oxidative stress in human islets, AIF was knocked down by small interfering RNAs (siRNA). Western blot analysis of AIF expression shows 76% downregulation of AIF protein expression in human islets exposed for 3 days to siRNA directed to AIF (siAIF) versus scrambled control (siScr, Fig. 3A). In line with our observations in islets from *Hq* mutant mice, exposure of isolated human islets to siAIF resulted in a 8.8-fold increase in β-cell apoptosis (*p*<0,05), analyzed by double staining for TUNEL and insulin (Fig. 3B).

After exposure to the siRNAs, isolated human islets were treated with 50 µM H_2_O_2_ for 2 h. This resulted in a 26-fold increase in β-cell apoptosis in the siScr islets and a 6.5-fold increase in the siAIF treated islets (Fig. 3B, *p*<0.05). Furthermore, H_2_O_2_-induced β-cell apoptosis was 2-fold higher in the AIF depleted islets compared to siScr control (*p*<0.05).

To assess the effect of AIF depletion on insulin secretion, we have performed a glucose stimulated insulin secretion assay (GSIS) in siAIF and siScr transfected human islets. Basal and stimulated insulin secretion (Fig. 3C) as well as the stimulatory index (Fig. 3D) was not significantly changed in response to AIF depletion. Despite its deleterious role in inducing β-cell apoptosis, AIF depletion did not impair β-cell function, neither basal nor glucose stimulated insulin secretion. Then we compared response to H_2_O_2_. As expected, H_2_O_2_ decreased glucose stimulated insulin secretion in control as well as in siAIF treated islets. Stimulatory index was decreased by 23% by H_2_O_2_ in siScr islets and by 40% in siAIF islets (p<0.05), assuming that AIF plays a role to protect the islets from oxidative stress, but is not involved in insulin secretion itself.

## Discussion

Recent work in *Hq* mutant mice, which have an 80% decrease in AIF expression, suggests that AIF depletion results in increased neuronal cell death [13]. Under physiologic conditions, AIF acts as a free radical scavenger in the mitochondrial respiratory chain to prevent apoptosis. Considering the high correlation of mitochondrial dysfunction and oxidative stress with T2DM, we hypothesized that AIF may play an important role in the regulation of β-cell mass and survival.

In the present study, we report that AIF depletion results in a 3-fold decrease in β-cell mass at 2-weeks of age which was further decreased with age. This was due to the missregulation of cell cycle control. Although there was no difference in proliferating β-cells in the Harlequin mice, 3-fold more β-cells in the *Hq* mice retained in G2 phase of the cell cycle. In accordance to previous studies we found that *Hq* β-cells are highly prone to apoptosis [21], [22].

A proposed mechanism for the induction of apoptosis could be oxidative stress, which is associated with abnormal cell cycle checkpoint function. Under normal circumstances the cell cycle proceeds without interruptions. However, if cycling cells are damaged by agents which induce oxidative stress, a G2 check point response is triggered and cells pause and repair the damage [24]. In the case of a severe damage, cells may alternatively undergo apoptosis. Given the role of AIF in mitochondrial respiration as a hydrogen peroxide scavenger, loss of AIF could result in an accumulation of ROS, which would then generate a misregulation of β-cell cycle and apoptosis. This is further supported by the finding, that β-cells from both AIF-deficient mice (*Hq* mutant mice) and human islets (AIF-siRNA-treated) displayed increased sensitive to cell death induced by hydrogen peroxide. β-cells are in general very sensitive to oxidative stress, in particular with hydrogen peroxide, due to their low expression of a peroxide scavenging system [7].

Given the role of β-cell mitochondria as key regulators of glucose stimulated insulin secretion, we hypothesized that *Hq* mice show an impaired glucose tolerance. Surprisingly, and in accordance to a recent study, AIF deficiency in *Hq* mutant mice did not impair glucose tolerance. *Hq* mice were rather hypoglycemic compared to wt. This could be due to impaired glucose sensing as a result of selective neuron loss reported in *Hq* mutant mice, but is more likely to be a result of improved insulin sensitivity [15]. Many studies have linked changes in mitochondrial oxidative phosphorylation to the development of insulin resistance and diabetes [14], [16], [25]: Importantly, a recent study in tissue specific muscle- and liver AIF knockout mice and *Hq* mice with a global AIF deficiency shows that a primary OxPhos defect alone does not cause diabetes, but rather increases insulin sensitivity and reduces fat mass [15]. In line with our results, this study found that *Hq* mice displayed improved glucose tolerance associated with reduced fasting and glucose-induced insulin secretion, possibly due to the improved insulin sensitivity. This explains, why *Hq* mice still maintain normoglycemia, despite their loss of β-cell mass. Interestingly, in the absence of oxidative stress, AIF depleted human islets did not show any changes in insulin secretion. When compared to WT islets, *Hq* islets showed a similar response to glucose, confirming the hypothesis that AIF does not directly act on insulin secretion. Nevertheless, when islets were exposed to H_2_O_2_, insulin secretion was further impaired in the AIF depleted islets, assuming that AIF plays a role in protecting the islets from oxidative stress, but is not involved in insulin secretion itself.

The proposed compensation mechanism involves a shift to anaerobic glucose metabolism and increase in fuel utilization [15]. It is important to note; that the *Hq* mice, despite their OxPhos deficiency, had no indication of elevated ROS levels [26]. However, our finding that AIF is necessary for maintaining β-cell mass is in favor of a critical role of ROS signaling and oxidative stress in the β-cells and supports the role of AIF as a free radical scavenger in the mitochondrial respiratory chain to prevent apoptosis.

Our data provide new insights into a protective effect of AIF on β-cell turnover. Loss of AIF could therefore be one of the mechanisms of β-cell apoptosis.

## References

[1] Donath MY, Halban PA (2004). Decreased beta-cell mass in diabetes: significance, mechanisms and therapeutic implications.. Diabetologia.

[2] Wiederkehr A, Wollheim CB (2006). Minireview: implication of mitochondria in insulin secretion and action.. Endocrinology.

[3] Wiederkehr A, Wollheim CB (2008). Impact of mitochondrial calcium on the coupling of metabolism to insulin secretion in the pancreatic beta-cell.. Cell Calcium.

[4] Krauss S, Zhang CY, Scorrano L, Dalgaard LT, St-Pierre J (2003). Superoxide-mediated activation of uncoupling protein 2 causes pancreatic beta cell dysfunction.. J Clin Invest.

[5] Maedler K, Spinas GA, Dyntar D, Moritz W, Kaiser N (2001). Distinct effects of saturated and monounsaturated fatty acids on beta-cell turnover and function.. Diabetes.

[6] Sakurai K, Katoh M, Someno K, Fujimoto Y (2001). Apoptosis and mitochondrial damage in INS-1 cells treated with alloxan.. Biol Pharm Bull.

[7] Maechler P, Jornot L, Wollheim CB (1999). Hydrogen peroxide alters mitochondrial activation and insulin secretion in pancreatic beta cells.. J Biol Chem.

[8] Maechler P, Wollheim CB (2001). Mitochondrial function in normal and diabetic beta-cells.. Nature.

[9] Susin SA, Lorenzo HK, Zamzami N, Marzo I, Snow BE (1999). Molecular characterization of mitochondrial apoptosis-inducing factor.. Nature.

[10] Cande C, Cohen I, Daugas E, Ravagnan L, Larochette N (2002). Apoptosis-inducing factor (AIF): a novel caspase-independent death effector released from mitochondria.. Biochimie.

[11] Liu X, Kim CN, Yang J, Jemmerson R, Wang X (1996). Induction of apoptotic program in cell-free extracts: requirement for dATP and cytochrome c.. Cell.

[12] Porter AG, Urbano AG (2006). Does apoptosis-inducing factor (AIF) have both life and death functions in cells?. Bioessays.

[13] Klein JA, Longo-Guess CM, Rossmann MP, Seburn KL, Hurd RE (2002). The harlequin mouse mutation downregulates apoptosis-inducing factor.. Nature.

[14] Lowell BB, Shulman GI (2005). Mitochondrial dysfunction and type 2 diabetes.. Science.

[15] Pospisilik JA, Knauf C, Joza N, Benit P, Orthofer M (2007). Targeted deletion of AIF decreases mitochondrial oxidative phosphorylation and protects from obesity and diabetes.. Cell.

[16] Rabol R, Boushel R, Dela F (2006). Mitochondrial oxidative function and type 2 diabetes.. Appl Physiol Nutr Metab.

[17] DiMauro S, Schon EA (2003). Mitochondrial respiratory-chain diseases.. N Engl J Med.

[18] Wallace DC (1999). Mitochondrial diseases in man and mouse.. Science.

[19] Hendzel MJ, Wei Y, Mancini MA, Van Hooser A, Ranalli T (1997). Mitosis-specific phosphorylation of histone H3 initiates primarily within pericentromeric heterochromatin during G2 and spreads in an ordered fashion coincident with mitotic chromosome condensation.. Chromosoma.

[20] Zhong L, Georgia S, Tschen SI, Nakayama K, Nakayama K (2007). Essential role of Skp2-mediated p27 degradation in growth and adaptive expansion of pancreatic beta cells.. J Clin Invest.

[21] Donath MY, Gross DJ, Cerasi E, Kaiser N (1999). Hyperglycemia-induced beta-cell apoptosis in pancreatic islets of Psammomys obesus during development of diabetes.. Diabetes.

[22] Ritzel RA, Butler PC (2003). Replication increases beta-cell vulnerability to human islet amyloid polypeptide-induced apoptosis.. Diabetes.

[23] van Empel VP, Bertrand AT, van der Nagel R, Kostin S, Doevendans PA (2005). Downregulation of apoptosis-inducing factor in harlequin mutant mice sensitizes the myocardium to oxidative stress-related cell death and pressure overload-induced decompensation.. Circ Res.

[24] Shackelford RE, Kaufmann WK, Paules RS (2000). Oxidative stress and cell cycle checkpoint function.. Free Radic Biol Med.

[25] Befroy DE, Petersen KF, Dufour S, Mason GF, de Graaf RA (2007). Impaired mitochondrial substrate oxidation in muscle of insulin-resistant offspring of type 2 diabetic patients.. Diabetes.

[26] Vahsen N, Cande C, Briere JJ, Benit P, Joza N (2004). AIF deficiency compromises oxidative phosphorylation.. Embo J.

