# Correction: Deletion of the Mitochondrial Flavoprotein Apoptosis Inducing Factor (AIF) Induces β-Cell Apoptosis and Impairs β-Cell Mass

**DOI:** 10.1371/journal.pone.0117766

**Published:** 2015-05-08

**Authors:** The PLOS ONE Staff

Panel 1 in Fig. 1A is incorrect. Please view a correct version of Fig. 1 here.

**Figure 1:**
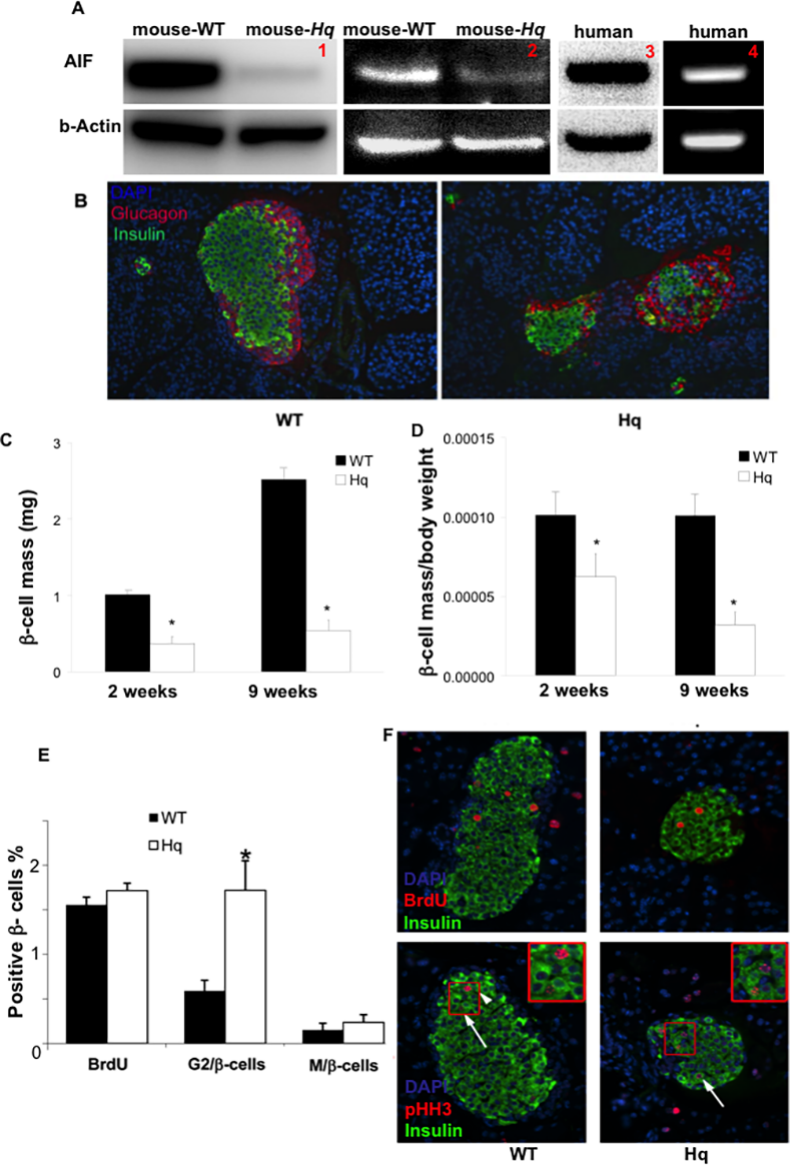
Decreased β-cell mass in Hq mutant mice. (A). Representative Western blots (panel 1,3) and PCR analyzes (panel 2,4) of AIF expression in isolated mouse (panel 1,2) and human (panel 3,4) islets. Actin was used as loading control/ house keeping gene. Western blots/ PCRs are representatives of three independent experiments from three mice or from 3 organ donors, respectively. (B) Histological analysis by insulin staining in green and glucagon staining in red show a normal islet cellular composition and smaller islets in 2-week-old *Hq* mutant mice. (C,D) Analysis of β-cell mass (C) or β-cell mass divided by body weight (D) of WT and*Hq* mutant mice at 2 and 9 weeks of age. Values are representative of 5 slides spanning the whole pancreas of each mouse and 4 mice for each group at each age (magnification x125). (E) Cell cycle characteristics of β-cells from WT mice and *Hq*mutant mice as measured by BrdU and pHH3 staining. BrdU^+^insulin^+^ cells are counted as β-cells at S phase (see example in F, upper panel). pHH3^+^ (with punctuated pattern) insulin+ cells are counted as β-cells at G2 phase (see example in F, Hq mice lower right panel). pHH3^+^ (with strong nuclear expression) insulin+ cells were counted as β-cells at M phase (see example in F, WT mice lower left panel). Data are shown as mean±SE. *P<0.05 in *Hq* mutant mice *vs*. WT mice.
