# Expression of Concern: Deletion of the Mitochondrial Flavoprotein Apoptosis Inducing Factor (AIF) Induces β-Cell Apoptosis and Impairs β-Cell Mass

**DOI:** 10.1371/journal.pone.0272901

**Published:** 2022-08-25

**Authors:** The PLOS ONE Editors

After publication of this article [1], concerns were raised about some of the western blots in Figs 1A and 3A. Specifically:

- The β-actin blot in section 1 of Fig 1A appears similar to lanes two and three in the actin blot in Fig 3G in [2].
- The mouse-WT band in the AIF blot in section 1 of Fig 1A appears similar to lane one in the actin blot in Fig 3G in [2], when the aspect ratio is adjusted.
- There appears to be a vertical discontinuity between the two β-actin lanes in Fig 3A.

Please note that article [2] has been retracted [3].

A previous correction notice for this article [1] was published on 8th May 2015 [4]. The correction provided a revised version of Fig 1A in which the mouse AIF and b-Actin panels were replaced, but the notice did not explain or clarify the issues with the original published figure. The *PLOS ONE* Editors apologize for this oversight.

The corresponding author stated that the leftmost b-Actin panel in Fig 1A in [1], which served as loading control for the protein lysates of isolated islets from wild-type and *Hq* mutant mice, did not report the correct blot for the experiment. The original blots underlying the panel in Fig 1A were no longer available at the time of the 2015 correction, so the authors provided another representative set of AIF and b-Actin blot from lysates of isolated islets of WT and *Hq* mice in support of the published results. The replication data in the corrected figure [2] support the claim that AIF expression is reduced in *Hq* mutant mice. The blot underlying panel 1 in the revised Fig 1A are in S1 File.

The corresponding author noted that the AIF and Actin bands in Fig 3A were spliced from their original position in the raw blots, in order to display them together. They stated that Fig 3A shows non-contiguous lanes from the same western blot and that the complete blot was not shown in article [1] because only the 50 nM concentration of siRNA used in the experiments was documented. The authors provide here a revised Fig 3 where the spliced bands are denoted by vertical white lines. Underlying data from the original experiment supporting Fig 3A are in S2–S4 Files, with the spliced out lanes demonstrated in S5 File.

The concern noted in the second bullet point, above, had not been identified at the time of the prior correction [4]. This issue remains unresolved and cannot be fully clarified due to the unavailability of the original image data.

Raw data underlying the remaining results reported in the article are available from the corresponding author.

Given the unavailability of the original data needed to clarify the concerns about Fig 1, concerns remain about data reporting in the original published version of this figure. Therefore, the *PLOS ONE* Editors issue this Expression of Concern.

The AIF and b-Actin panels reporting mouse tissue western blot experiments in Fig 1A appear similar to content in [2], published in 2006 by the American Diabetes Association and which is not offered under a CC-BY license. These two panels of [1] are therefore excluded from the *PLOS ONE* article’s [1] license. At the time of this notice’s publication, the article [1] was republished to note this exclusion in the Fig 1 legend and the article’s copyright statement.

**Fig 3:**
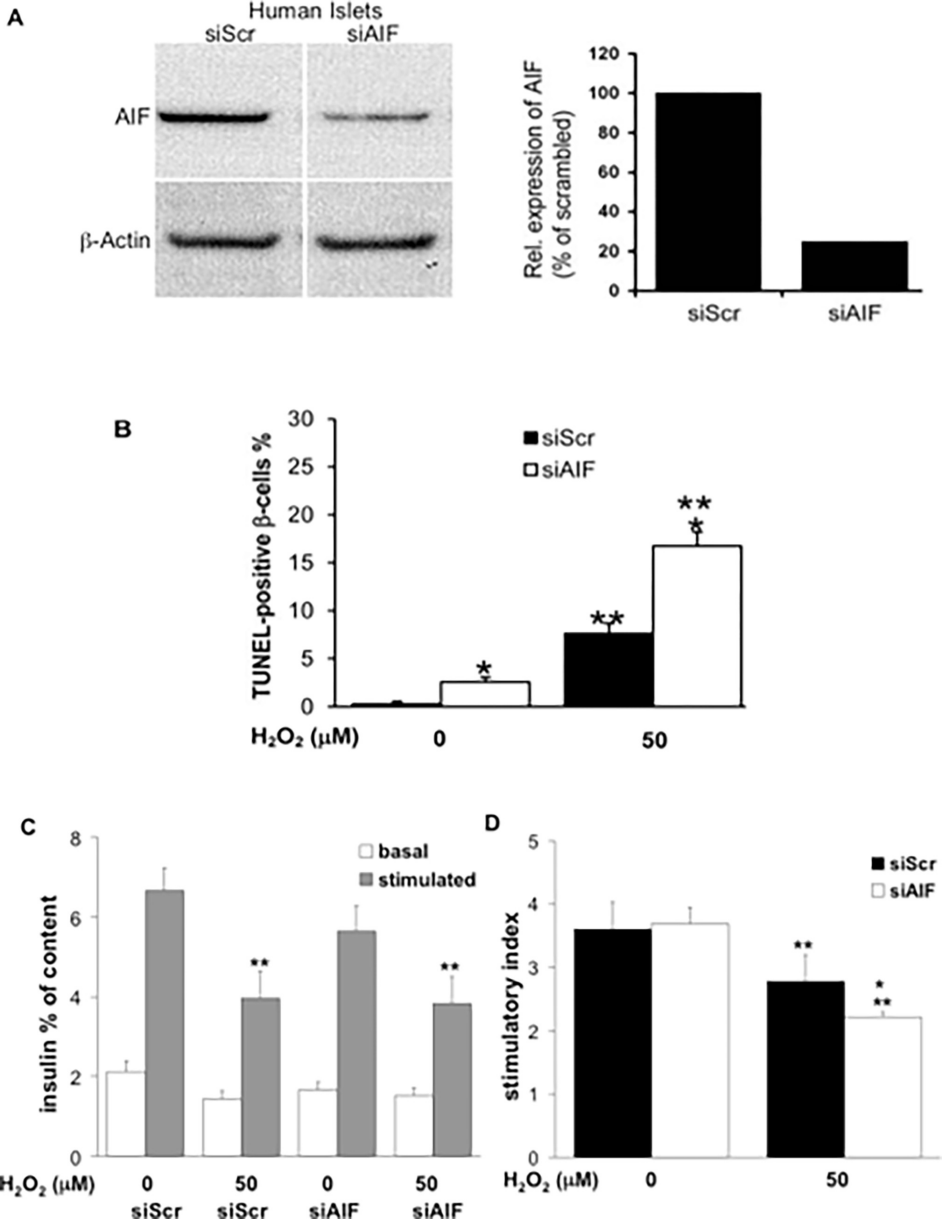
AIF depletion leads to increased β-cell apoptosis in human islets without affecting insulin secretion. (A) Isolated human pancreatic islets were exposed to siRNA to AIF (siAIF) or scrambled control siRNA (siScr) for 3 days. The knockdown efficiency was determined by Western blot analysis. Actin was used as a loading control on the same membrane after stripping. This Western blot is representative of three independent experiments from three different organ donors. The density of expression levels were quantified after scanning and normalised to actin levels. (B–D) 3 days after transfection, islets were exposed to 50 μM H_2_O_2_ for 2 h. (B) Islet sections were prepared for analysis of β-cell apoptosis by the TUNEL assay. Islets were double-stained for insulin in green and counterstained for DAPI in blue. Results are means ± SE of the percentage of TUNEL-positive β-cells. The mean number of β-cells counted was 3400 for each treatment condition. (C,D) GSIS: after the H_2_O_2_ treatment, islets were washed and basal and stimulated insulin secretion analyzed during successive 1-h incubations at 2.8 mM (basal) and 16.7 mM (stimulated) glucose. Data are normalized to insulin content. (D) Stimulatory index denotes the ratio between stimulated and basal values of insulin secretion. (B–D) All assays were performed in triplicate or quadruplicate in three independent experiments from 3 different organ donors, respectively. *p<0.05 to siScr control, **p<0.05 in H_2_O_2_ treated vs. untreated control.

## Supporting information

S1 File Data underlying panel 1 in the revised Fig 1A. (PDF)

S2 File Data underlying the AIF panel in Fig 3A. (TIF)

S3 File Data underlying the b-action panel in Fig 3A. (TIF)

S4 File Data underlying the chart in Fig 3A. (XLS)

S5 File The construction of Fig 3A. (PPTX)
