# Supplementary material for: Expression of Concern: Deletion of the Mitochondrial Flavoprotein Apoptosis Inducing Factor (AIF) Induces β-Cell Apoptosis and Impairs β-Cell Mass
Source: PLoS One. 2022 Aug 25;17(8):e0272901. doi: 10.1371/journal.pone.0272901 (PMC9409564; doi:10.1371/journal.pone.0272901)
Supplement: S1 File — (PDF) [file pone.0272901.s001.pdf]

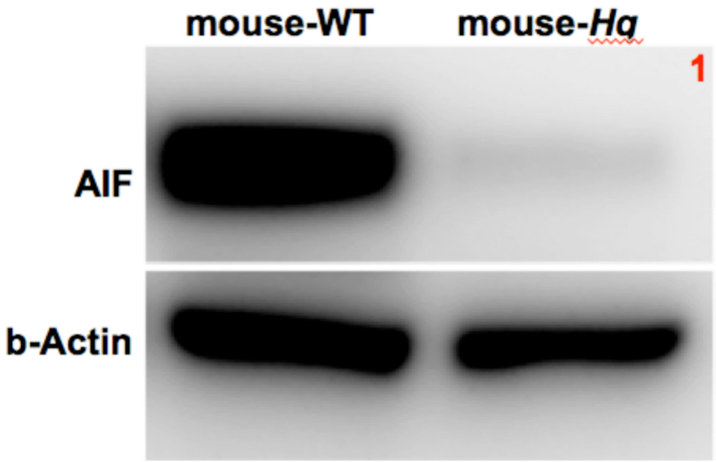

Figure 1, panel 1: Representative western blot of AIF expression in isolated mouse islets from 3-month-old WT and *Hq* mice.

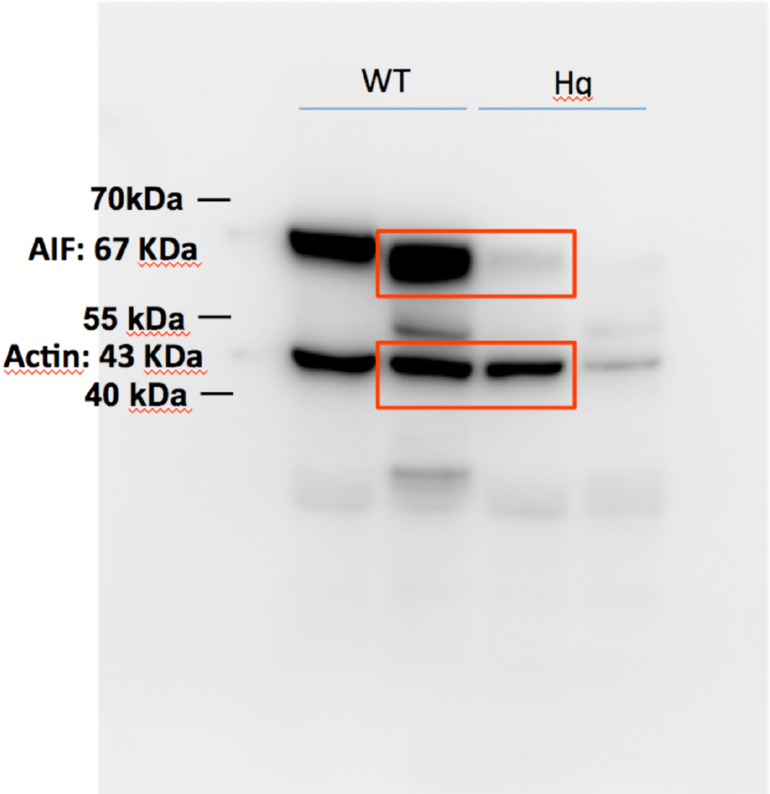

FULL BLOT from 2 islet isolations from WT & Hq mice, respectively.
