# Supplementary figures and images for: Expression of Concern: Deletion of the Mitochondrial Flavoprotein Apoptosis Inducing Factor (AIF) Induces β-Cell Apoptosis and Impairs β-Cell Mass
Source: PLoS One. 2022 Aug 25;17(8):e0272901. doi: 10.1371/journal.pone.0272901 (PMC9409564; doi:10.1371/journal.pone.0272901)

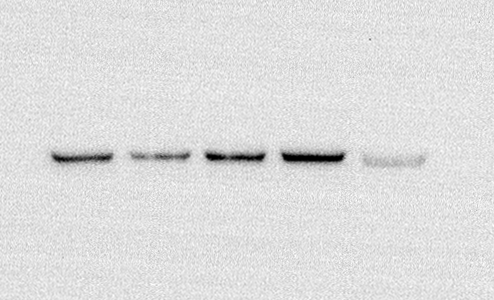

Supplement: S2 File — (TIF) [file pone.0272901.s002.tif]

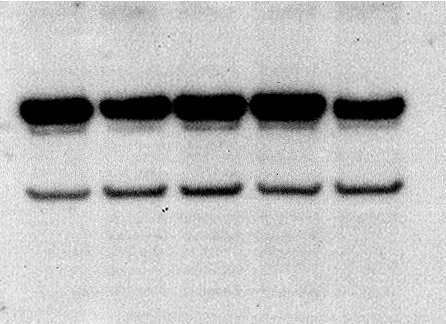

Supplement: S3 File — (TIF) [file pone.0272901.s003.tif]

## Slide 1
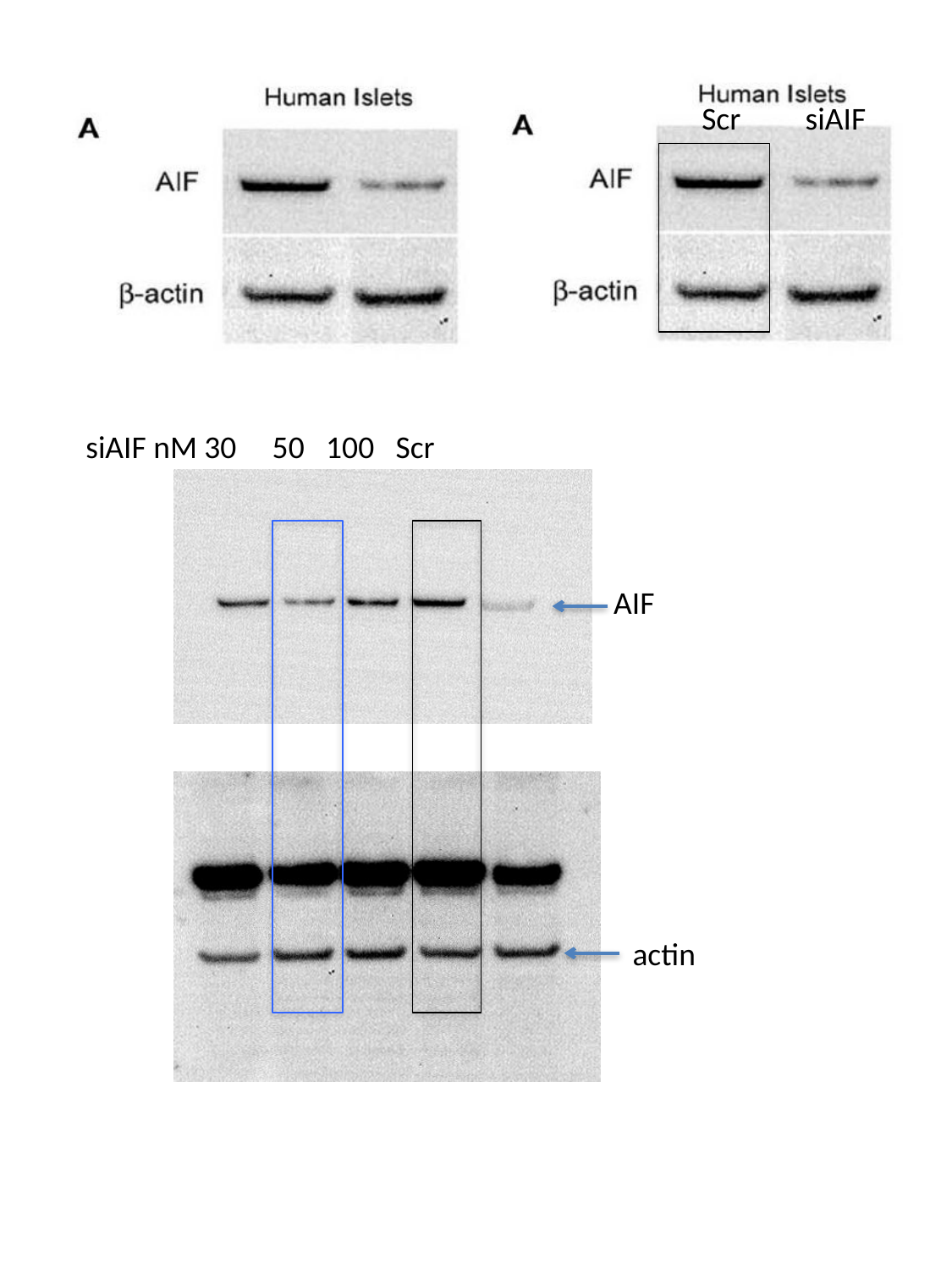

siAIF nM 30 50 100 Scr
AIF
actin
Scr
siAIF
Scr
siAIF

Supplement: S5 File — (PPTX) [file pone.0272901.s005.pptx]
